# Supplementary material for: Resolving the full spectrum of human genome variation using Linked-Reads
Source: Genome Res. 2019 Apr;29(4):635–45. doi: 10.1101/gr.234443.118 (PMC6442396; doi:10.1101/gr.234443.118)
Supplement: Supplemental Material [file supp_gr.234443.118_Supplemental_longranger-2.2.zip › longranger-2.2.tar.bz2!/lr2.2-public/lib/go/src/code.google.com/vitess/vitess.io/_includes/page-author.html]

{% if page.author %}
{% assign author = site.data.authors[page.author] %}{% else %}{% assign author = site.owner %}
{% endif %}

### Written by {% if author.web %}{{ author.name }}{% else %}{{ author.name }}{% endif %}

{{ author.bio }}
